# Structural Modeling on the Determinants of Effectiveness of SOPs Containing COVID-19 in Mass Gatherings

**DOI:** 10.3389/fpsyg.2021.755221

**Published:** 2021-10-27

**Authors:** Abdul Basit, Miklas Scholz, Abdul Aziz Khan Niazi, Tehmina Fiaz Qazi, Muhammad Zeeshan Shaukat, Zia-ur-Rehman Rao, Asif Mahmood

**Affiliations:** ^1^Lahore Institute of Science and Technology, Lahore, Pakistan; ^2^Division of Water Resources Engineering, Department of Building and Environmental Technology, Faculty of Engineering, Lund University, Lund, Sweden; ^3^Department of Civil Engineering Science, School of Civil Engineering and the Built Environment, University of Johannesburg, Johannesburg, South Africa; ^4^Department of Town Planning, Engineering Networks and Systems, South Ural State University (National Research University), Chelyabinsk, Russia; ^5^Institute of Business and Management, University of Engineering and Technology, Lahore, Pakistan; ^6^Hailey College of Banking and Finance, University of the Punjab, Lahore, Pakistan; ^7^Faculty of Management Studies, University of Central Punjab, Lahore, Pakistan; ^8^Hailey College of Commerce, University of the Punjab, Lahore, Pakistan; ^9^Department of Business Studies, Namal Institute, Mianwali, Pakistan

**Keywords:** COVID-19, determinants, ISM, mass gathering, MICMAC, SOPs

## Abstract

The study is aimed to analyze the determinants of the effectiveness of SOPs in mass gatherings for containing COVID-19. The overall design of the study involves a literature review, data collection by field survey, structural modeling, and analysis. The study is built on the experts' opinion of a focus group (representing people who recently participated in and are responsible for mass gatherings). The study uses the discussion of the literature review to identify the determinants, interpretive structural modeling (ISM) for developing and analyzing a structural model, and Matrice d'Impacts Croises Multiplication Appliquée a un Classement (MICMAC) for corroboration of results of the ISM/classification of determinants. From the literature review, a list of determinants is generated and verified by a panel of experts. The results of the ISM revealed that the determinants “legal environment of the country,” “practicability of SOPs,” “perceived benefit of adapting SOPs,” and “possibilities of avoiding gathering” occupied the top of the model, therefore, they are less critical determinants, whereas “nature of gathering” occupied the bottom of the model, and is thus the most critical determinant. The remaining determinants form the middle of the model, and are therefore moderately severe. The results of MICMAC show that the determinant “perceived benefit of adapting SOPs” is dependent, “nature of gathering” is independent, and all others are linked. The results of MICMAC implicitly substantiate the findings of the ISM. The overall results of the study show that “nature of gathering” is the key determinant. This research does not require a priori theory since it is a theory-building study that uses an inductive approach. It is based on real data and it is useful for local authorities, organizers, participants (attendees/visitors) of mass gatherings, health officials/regulators, researchers, and the community at large. This study has fundamental importance for planning and preparing for such events while ensuring the minimum risk of COVID-19 transmission.

## Introduction

COVID-19 has wrought havoc around the globe, with 200,840,180 confirmed infected cases and 4,265,903 confirmed deaths worldwide (World Health Organization, 2021). The pandemic has pushed mankind to reshape the global order (Akon and Rahman, 2020; Rehman and Ahmad, 2020). The outbreak of the COVID-19 pandemic in 2019 (now considered as *coronaviridae* family) has redirected the thinking of policymakers. It also has forced governments and communities to revisit governance, social, and business practices. Discouragement of gathering and social mixing has emerged as one of the measures to contain the transmission of novel disease. Evaluation of this precaution, qua reality, has gained fundamental importance because in certain cases, social distancing and its SOPs are practicable, but in certain cases it is difficult if not impossible. Particularly in mass gatherings, the practicability of the SOPs of social distancing is highly questionable. Mass gatherings are events that are capable of amplifying the transmission of COVID-19 and potentially disrupt the country's response capacity to the COVID-19 pandemic. Mass gathering includes cultural events, religious gatherings, sports events, entertainment concerts, political procession/assemblies, congregations, and crowds. The gatherings may be indoor or outdoor, since as far as the SOPs are concerned they are questionable in both cases. It has become highly important to analyze and uncover the structures of the relationships of the determinants of the effectiveness of SOPs of social distancing to contain COVID-19, particularly in mass gatherings. Right from the outbreak of this disease, researchers started attempting to make some sense of the system. For example, Carlin et al. (2021) evaluated the impact of the COVID-19 pandemic on mass gatherings for hockey and basketball games in North America, Hughes et al. (2020) conducted a study on the framework of sports in Australia, Mat et al. (2020) evaluated and found COVID-19 contamination in Malaysia because of mass gatherings, Nyasulu et al. (2021) argued that the COVID-19 outbreak emerged in Malawi-Southeastern Africa as a result of sociopolitical mass gatherings, Teixeira (2020) highlighted a possible transmission of COVID-19 due to mass gatherings in Brazil, Yezli and Khan (2020) highlighted possible health risks from the transmission of COVID-19 due to mass gatherings in Jeddah-KSA. A lot of research has also been conducted on religious mass gatherings and religious tourism with the perspective of COVID-19, including but not limited to the studies of Ahmed and Memish (2020), Atique and Itumalla (2020), Ebrahim and Memish (2020), Hoang et al. (2020), Mat et al. (2020), Mubarak (2020), Mubarak and Zin (2020), Quadri (2020), Rodriguez-Morales et al. (2020), VanderWeele (2020), Yezli and Khan (2020), Hashim et al. (2021), Hsu et al. (2021), Sokhna et al. (2021), and Yezli and Khan (2021). Although some of these studies address the issue partially or indirectly, there is still a clear gap in the literature regarding real determinants of the implementation of SOPs for COVID-19 in mass gatherings. This study is designed to fill this gap by way of analyzing the determinants of the effectiveness of SOPs in mass gatherings for containing COVID-19. The objectives of the study are: (i) to identify the determinants of the effectiveness of the SOPs of containing COVID-19, (ii) to develop and analyze a structural model of the determinants, (iii) to corroborate the results of the structural model, and (iv) to classify the determinants. For achieving these objectives, an array of methodologies was considered (Shaukat et al., 2021) and interpretive structural modeling (ISM) augmented with Matrice d'Impacts Croises Multiplication Appliquée a un Classement (MICMAC) was chosen as the methodology. It has many advantages over its rival methodologies. It is an exploratory approach, appropriate for making some sense of the early stages of systems. It takes a smaller amount of data and converts fragile and unclear mental models into graphical models to simplify complex issues. It outperforms the statistical methodologies that take large statistical data but contribute little toward the literature. The rest of this study is divided into the Literature Survey, the Methodology, the Modeling, Analysis, Results, and Discussion, and the Conclusion.

## Literature Survey

A survey of the literature is important: to set out the context of the study, to account for the current literature, and to acknowledge contemporary work. A thorough survey of the literature was conducted. We explored the online databases of Wiley-Blackwell, JStor, Emerald, Springerlink, Sage, Taylor and Francis, Elsevier (Science Direct), etc. Keywords used for the search include: mass gathering, COVID-19, social distancing, SOPs of COVID-19, coronavirus disease, novel disease of coronavirus, determinants of SOPs of COVID-19, social distancing, etc. An influx of published/unpublished literature is found to be available online. A large number of articles were also found as “accepted for publication.” Hundreds of relevant papers were reviewed, and only a small amount of literature was found directly concerning the issue. It is not out of place to report some of the studies that can provide foundations for understanding the current phenomenon. McCloskey et al. (2020) proclaimed that the banning of mass gathering events could result in plummeting further global spread of COVID-19. Memish et al. (2020) concluded that the COVID-19 epidemic demands internationally combined, lucid, and cooperative measures by communities, individuals, institutions, commercial bodies, and regulators to suspend gatherings and mitigate the transmission. Nyasulu et al. (2021) has argued that mass anti-government demonstrations and cross-border immigration are causing increased COVID-19 disease and mortality, particularly among people aged 50–59 years. Piovani et al. (2021) studied the longitudinal data of 37 countries to analyze the effect of banning mass gatherings on COVID-19 cumulative mortality. The results affirmed that a 1-day delay in exercising the banning of mass gatherings is associated with an increase in mortality of 6.97%. Chakraborty and Maity (2020) asserted that COVID-19 has had a devastating impact on society which could be controlled by restricting mass gatherings. Through our review of the literature, an initial list of determinants (Table 1) of the effectiveness of the SOPs for mass gatherings for containing COVID-19 was prepared and placed before a panel of experts for ratification.

**Table 1:** List of determinants of effectiveness of SOPs for containing COVID-19.

**Code**	**Determinants**	**Description**	**Source**
**1**	Nature of gathering	The category of gathering e.g., cultural, religious, sports, political, etc.	Memish et al. (2019)
**2**	Type of gathering community	The class of people interested/required to participate in the gathering.	Ebrahim and Memish (2020)
**3**	Available physical space at gathering place	Allocated physical space at gathering place to the gathering.	Suggested by experts
**4**	Existence of penal clauses in SOPs	Existence of penal clauses in rules/regulations/guidelines and/or law whatsoever regarding not observing the SOPs to prevent coronavirus.	Al-Tawfiq et al. (2021)
**5**	Campaign of awareness regarding SOPs	An organized course of action to create awareness among the people regarding SOPs to prevent coronavirus.	Alshammari et al. (2020); Sonis et al. (2020)
**6**	Legal environment of the country	Laws made by the government for preventing the people from coronavirus and its seriousness.	Sokhna et al. (2021)
**7**	Practicability of SOPs	Practical possibilities of observing the SOPs in a real-life situation.	Furuse (2021a)
**8**	Perceived benefit of adapting SOPs	Perception of the people about the benefits of SOPs qua reality.	Furuse (2021b)
**9**	Possibilities of implementation of penal clauses	The likelihood or chance of the implementation of penal clauses if at all existing or incorporated in the relevant law.	Al-Tawfiq et al. (2021)
**10**	Possibilities of avoiding gathering	How much is it practically possible for a person to avoid gathering?	Ahmed and Memish (2020)
**11**	Seriousness of SOPs implementers	How much is government or implementers (whatsoever) serious in implementing the SOPs?	Hsu et al. (2021)

For the verification of the initial list of determinants, a panel of experts consisting of 10 Ph.D. doctors was constituted. The experts are individually approached and asked to (i) evaluate the determinants, (ii) add determinants if they were not already on the list, (iii) merge two or more determinants if appropriate, (iv) delete determinants that seem irrelevant, (v) modify determinants if necessary, and (vi) vote for the inclusion of determinants in the study. The experts added one of the determinants (available physical space at gathering place). Although the determinants have been gathered from the literature, it is still important to get our findings ratified by a panel of experts because the literature collected is diverse, meager, and assorted. Anecdotal evidence of the determinants demands that we verify qua relevance, appropriateness, and sufficiency. In this way, the list of determinants is finalized for inclusion in the study (Table 2).

**Table 2:** Voting sheet.

**Sr**.	**Determinants**	**Experts**										**Vote count**		**Decision**
		**1**	**2**	**3**	**4**	**5**	**6**	**7**	**8**	**9**	**10**	**Yes**	**No**	
**1**	Nature of gathering	***√***	***√***	*X*	***√***	***√***	***√***	***√***	***√***	*X*	***√***	8	2	Included
**2**	Type of gathering community	***√***	***√***	***√***	***√***	***√***	***√***	***√***	***√***	*X*	***√***	9	1	Included
**3**	Available physical space at gathering place	***√***	***√***	***√***	***√***	*X*	***√***	***√***	***√***	***√***	***√***	9	1	Included
**4**	Existence of penal clauses in SOPs	*X*	***√***	*X*	***√***	*X*	*X*	***√***	***√***	***√***	*X*	5	5	Included
**5**	Campaign of awareness regarding SOPs	***√***	***√***	***√***	***√***	***√***	***√***	***√***	***√***	***√***	***√***	10	0	Included
**6**	Legal environment of the country	*X*	***√***	*X*	***√***	***√***	***√***	*X*	***√***	***√***	*X*	6	4	Included
**7**	Practicability of SOPs	***√***	***√***	***√***	***√***	***√***	*X*	***√***	***√***	***√***	***√***	9	1	Included
**8**	Perceived benefit of adapting SOPs	***√***	***√***	***√***	***√***	***√***	*X*	***√***	***√***	***√***	***√***	9	1	Included
**9**	Possibilities of implementation of penal clauses	***√***	***√***	***√***	***√***	***√***	***√***	*X*	***√***	***√***	***√***	9	1	Included
**10**	Possibilities of avoiding gathering	***√***	***√***	***√***	***√***	***√***	***√***	*X*	***√***	***√***	***√***	9	1	Included
**11**	Seriousness of SOPs implementers	***√***	***√***	***√***	***√***	***√***	***√***	***√***	***√***	***√***	***√***	10	0	Included

In the case of determinant 4 (“existence of penal clauses in SOPs,” marked gray in Table 2) a tie occurred in the voting of the panel of experts. The authors, therefore, themselves evaluated the determinant and decided to include it in the study since it has fundamental importance and logically qualifies as part of the phenomenon under study.

## Methodology

This work is an exploratory inductive study which embraces a literature review, data collection survey, structural modeling, and analysis. The methodology of the study focuses on the discussion of the literature review for the identification of the determinants, ISM for developing and analyzing a structural model, and MICMAC for the corroboration of the results of the ISM/classification of determinants. Interpretive structural modeling is very useful a tool for qualitative analysis. It is appropriate for analyzing complex interdependent relationships in rapidly changing and entangled situations. It is capable of articulating complexities into visible, well-defined models with graphical presentations (Warfield, 1974; Sushil, 2012; Li et al., 2019). Since the COVID-19 pandemic is a new and complex phenomenon that is being explored, the method is not only justified but also the most appropriate. It outperforms statistical approaches in the cases like the one at hand. The population of the study consists of the people participating or responsible for mass gatherings. The sampling design of the study is based on a non-probability type of sampling that is a focus group (panel of experts) from within the population. The sample has been drawn on the basis of pre-determined criteria. The size of the sample consists of 10 experts on a panel. There are various techniques for finalizing the variables to be included in the investigation. Common techniques used include a literature review or expert opinion (Majumdar and Sinha, 2019), case study (Li et al., 2019), Delphi method (Bhosale and Kant, 2016), exploratory factor analysis (Li and Yang, 2014), presumption of the authors or meta-analysis (Lohaus and Habermann, 2019), idea engineering workshop and brainstorming session (Kumar et al., 2013), and interview content analysis (Xiao, 2018). This study used a literature review because this method has advantages over its rivals. It provides a foundation of knowledge about the phenomenon under study, prevents duplication, gives credit to other researchers, highlights questions left from other research, provides justification of inclusion or exclusion of the variables in the study, and indicates the potential relationships among variables sets at the outset of the study. The data have been collected from the experts using a matrix type questionnaire that contains precise instructions for respondents about completing the questionnaire. The respondents were instructed as follows: (i) fill only white cells (*ij* part only) and do not fill black and gray cells (Table A1 in Appendix), (ii) contextual relationship = leads to, (iii) enter *V* when the row leads to the column, (iv) enter *A* when the column leads to the row, (v) enter *O* when there is no relation between the row and the column, and (vi) enter *X* when row and column lead to each other. The study uses the classical form of ISM that progresses stepwise and develops a hierarchical structural model by way of exploiting the binary matrices. It also uses MICMAC for the classification of the determinants and the analysis of their driving power and dependence power on a Cartesian plane.

### Panel of Experts

To generating the primary data, a panel of experts was constituted according to the norms of ISM studies. Panels are constituted where the data does not exist, data collection is expensive, available data is limited, and/or the data is unreliable. This is the case in our study, therefore, we find it appropriate to form a panel of experts as a source of eliciting the primary data. This panel of respondents is different from the aforementioned panel used for the verification of the factors. There are two types of respondent panel i.e., homogeneous and heterogeneous. The size of the panel depends on the type of panel. In the current study, we intended to collect data from people responsible for and/or participating in different types of gathering, the context demands a heterogeneous panel. Therefore, the study uses a heterogeneous panel. The usual size of a heterogeneous panel varies from 8 to 12 experts. In this study, the panel of experts consisted of 10 experts (Jena et al., 2017). When recruiting experts for a panel, theoretical knowledge of the phenomenon under study, expert knowledge, and practical experience are the key criteria. In this case, the experts were recruited on the basis of their knowledge and recent experience of some familial, political, religious, funereal, or accidental mass gathering. Apart from this criterion, all the respondents are well-educated (minimum university graduates) and held responsible positions within organization, family, and society. The experts were identified, a rapport was developed with them, then they were invited to participate in this study and asked to fill a questionnaire. It took us more than 2 months to complete this process. There are different methods to elicit the primary data from the minds of the people. These methods include Delphi, brainstorming, nominal group technique, repertory-grid interview technique, matrix type questionnaire, laddering interview, one-to-one, face-to-face in-depth interview, triadic sorting, and elect alternatives (*VAXO*) for every pair of relations (Niazi et al., 2019; Qazi et al., 2020). This study used the one-to-one, face-to-face interview approach coupled with elect alternatives (*VAXO*) for every pair of relations. The data were collected individually from each expert in the *ij* part of the matrix using *VAXO* symbols. The data was aggregated in MS Excel sheet applying the “count if” function and “majority rule” in the case of every pair. This panel was involved at two stages: stage one at data collection and stage two at model verification. After developing the model, the panel of experts was approached again to review the structural model logically, theoretically, and conceptually.

## Modeling, Analysis, Results, and Discussion

### ISM Model Building

From the aggregated data, a structural self-interaction matrix (SSIM) is developed (Table 3).

**Table 3:** Structural self-interaction matrix.

**Code**	**Description**	**1**	**2**	**3**	**4**	**5**	**6**	**7**	**8**	**9**	**10**	**11**
**1**	Nature of gathering		V	V	V	V	V	V	V	V	V	V
**2**	Type of gathering community			V	A	A	A	A	V	V	V	V
**3**	Available physical space at gathering place				A	A	V	V	V	V	V	V
**4**	Existence of penal clauses in SOPs					A	A	A	V	V	V	V
**5**	Campaign of awareness regarding SOPs						A	V	V	A	V	V
**6**	Legal environment of the country							A	V	V	A	V
**7**	Practicability of SOPs								V	V	A	X
**8**	Perceived benefit of adapting SOPs									A	V	A
**9**	Possibilities of implementation of penal clauses										V	A
**10**	Possibilities of avoiding gathering											A
**11**	Seriousness of SOPs implementers											

The SSIM is converted into an initial reachability matrix (Table 4) by applying the classical rules for converting an SSIM into binary codes.

**Table 4:** Initial reachability matrix.

**Code**	**Description**	**1**	**2**	**3**	**4**	**5**	**6**	**7**	**8**	**9**	**10**	**11**
**1**	Nature of gathering	1	1	1	1	1	1	1	1	1	1	1
**2**	Type of gathering community	0	1	1	0	0	0	0	1	1	1	1
**3**	Available physical space at gathering place	0	0	1	0	0	1	1	1	1	1	1
**4**	Existence of penal clauses in SOPs	0	1	1	1	0	0	0	1	1	1	1
**5**	Campaign of awareness regarding SOPs	0	1	1	1	1	0	1	1	0	1	1
**6**	Legal environment of the country	0	1	0	1	1	1	0	1	1	0	1
**7**	Practicability of SOPs	0	1	0	1	0	1	1	1	1	0	1
**8**	Perceived benefit of adapting SOPs	0	0	0	0	0	0	0	1	0	1	0
**9**	Possibilities of implementation of penal clauses	0	0	0	0	1	0	0	1	1	1	0
**10**	Possibilities of avoiding gathering	0	0	0	0	0	1	1	0	0	1	0
**11**	Seriousness of SOPs implementers	0	0	0	0	0	0	1	1	1	1	1

Every 0 on the initial reachability matrix is evaluated from the view point of transitive relations and on the basis of evidence of transitivity, some of the 0 s are replaced with 1^*^, and the reachability matrix is revised to form the final reachability matrix (fully transitive reachability matrix) Table 5.

**Table 5:** Final reachability matrix.

**Code**	**Description**	**1**	**2**	**3**	**4**	**5**	**6**	**7**	**8**	**9**	**10**	**11**	**Driving**
**1**	Nature of gathering	1	1	1	1	1	1	1	1	1	1	1	**11**
**2**	Type of gathering community	0	1	1	0	1^*^	1^*^	1^*^	1	1	1	1	**9**
**3**	Available physical space at gathering place	0	1^*^	1	1^*^	1^*^	1	1	1	1	1	1	**10**
**4**	Existence of penal clauses in SOPs	0	1	1	1	1^*^	1^*^	1^*^	1	1	1	1	**10**
**5**	Campaign of awareness regarding SOPs	0	1	1	1	1	1^*^	1	1	1^*^	1	1	**10**
**6**	Legal environment of the country	0	1	1^*^	1	1	1	1^*^	1	1	1^*^	1	**10**
**7**	Practicability of SOPs	1^*^	1	1^*^	1	1^*^	1	1	1	1	1^*^	1	**11**
**8**	Perceived benefit of adapting SOPs	0	0	0	0	0	1^*^	1^*^	1	0	1	0	**4**
**9**	Possibilities of implementation of penal clauses	0	1^*^	1^*^	1^*^	1	1^*^	1^*^	1	1	1	1^*^	**10**
**10**	Possibilities of avoiding gathering	0	1^*^	0	1^*^	1^*^	1	1	1^*^	1^*^	1	1^*^	**9**
**11**	Seriousness of SOPs implementers	0	1^*^	0	1^*^	1^*^	1^*^	1	1	1	1	1	**9**
**Dependence**		**2**	**10**	**8**	**9**	**10**	**11**	**11**	**11**	**10**	**11**	**10**	

* *is used to maintain distinction between direct relations and transitive relations*.

The final reachability matrix is partitioned through the iteration method, exploiting the elementary concepts of set theory (Warfield, 1973, 1974). The iterations have been performed following the classical rules of iteration (Warfield, 1973, 1974) and presented below in Tables 6–9.

**Table 6:** Iteration I.

**Code**	**Reachability set**	**Antecedent set**	**Intersection set**	**Level**
**1**	1,2,3,4,5,6,7,8,9,10,11	1,7	1,7	
**2**	2,3,5,6,7,8,9,10,11	1,2,3,4,5,6,7,9,10,11	2,3,5,6,7,9,10,11	
**3**	2,3,4,5,6,7,8,9,10,11	1,2,3,4,5,6,7,9	2,3,4,5,6,7,9	
**4**	2,3,4,5,6,7,8,9,10,11	1,3,4,5,6,7,9,10,11	3,4,5,6,7,9,10,11	
**5**	2,3,4,5,6,7,8,9,10,11	1,2,3,4,5,6,7,9,10,11	2,3,4,5,6,7,9,10,11	
**6**	2,3,4,5,6,7,8,9,10,11	1,2,3,4,5,6,7,8,9,10,11	2,3,4,5,6,7,8,9,10,11	*I*
**7**	1,2,3,4,5,6,7,8,9,10,11	1,2,3,4,5,6,7,8,9,10,11	1,2,3,4,5,6,7,8,9,10,11	*I*
**8**	6,7,8,10	1,2,3,4,5,6,7,8,9,10,11	6,7,8,10	*I*
**9**	2,3,4,5,6,7,8,9,10,11	1,2,3,4,5,6,7,9,10,11	2,3,4,5,6,7,9,10,11	
**10**	2,4,5,6,7,8,9,10,11	1,2,3,4,5,6,7,8,9,10,11	2,4,5,6,7,8,9,10,11	*I*
**11**	2,4,5,6,7,8,9,10,11	1,2,3,4,5,6,7,9,10,11	2,4,5,6,7,9,10,11	

**Table 7:** Iteration II.

**Code**	**Reachability set**	**Antecedent set**	**Intersection set**	**Level**
**1**	1,2,3,4,5,9,11	1	1	
**2**	2,3,5,9,11	1,2,3,4,5,9,11	2,3,5,9,11	*II*
**3**	2,3,4,5,9,11	1,2,3,4,5,9	2,3,4,5,9	
**4**	2,3,4,5,9,11	1,3,4,5,9,11	3,4,5,9,11	
**5**	2,3,4,5,9,11	1,2,3,4,5,9,11	2,3,4,5,9,11	*II*
**9**	2,3,4,5,9,11	1,2,3,4,5,9,11	2,3,4,5,9,11	*II*
**11**	2,4,5,9,11	1,2,3,4,5,9,11	2,4,5,9,11	*II*

**Table 8:** Iteration III.

**Code**	**Reachability set**	**Antecedent set**	**Intersection set**	**Level**
**1**	1,3,4	1	1	
**3**	3,4	1,3,4	3,4	*III*
**4**	3,4	1,3,4	3,4	*III*

**Table 9:** Iteration IV.

**Code**	**Reachability set**	**Antecedent set**	**Intersection set**	**Level**
**1**	1	1	1	*IV*

Hierarchies that were determined at different levels as a result of the iterations were extracted in a conical matrix and a digraph was prepared but, being an optional step in the ISM procedure, not reported here for brevity. The ISM process is presented in a condensed form as Table 10.

**Table 10:** Condensed presentation of ISM.

	**Reachability sets**														
	**Level**	**Code**	**6**	**7**	**8**	**10**	**2**	**5**	**9**	**11**	**3**	**4**	**1**		
**Antecedent sets**	**I**	**6**	1	1^*^	1	1^*^	1	1	1	1	1^*^	1	0	**11**	**Driving power**
		**7**	1	1	1	1^*^	1	1^*^	1	1	1^*^	1	1^*^	**9**	
		**8**	1^*^	1^*^	1	1	0	0	0	0	0	0	0	**10**	
		**10**	1	1	1^*^	1	1^*^	1^*^	1^*^	1^*^	0	1^*^	0	**10**	
	**II**	**2**	1^*^	1^*^	1	1	1	1^*^	1	1	1	0	0	**10**	
		**5**	1^*^	1	1	1	1	1	1^*^	1	1	1	0	**10**	
		**9**	1^*^	1^*^	1	1	1^*^	1	1	1^*^	1^*^	1^*^	0	**11**	
		**11**	1^*^	1	1	1	1^*^	1^*^	1	1	0	1^*^	0	**4**	
	**III**	**3**	1	1	1	1	1^*^	1^*^	1	1	1	1^*^	0	**10**	
		**4**	1^*^	1^*^	1	1	1	1^*^	1	1	1	1	0	**9**	
	**IV**	**1**	1	1	1	1	1	1	1	1	1	1	1	**9**	
			**2**	**10**	**8**	**9**	**10**	**11**	**11**	**11**	**10**	**11**	**10**		
**Dependence power**															

* *is used to maintain distinction between direct relations and transitive relations*.

**Figure 1:**
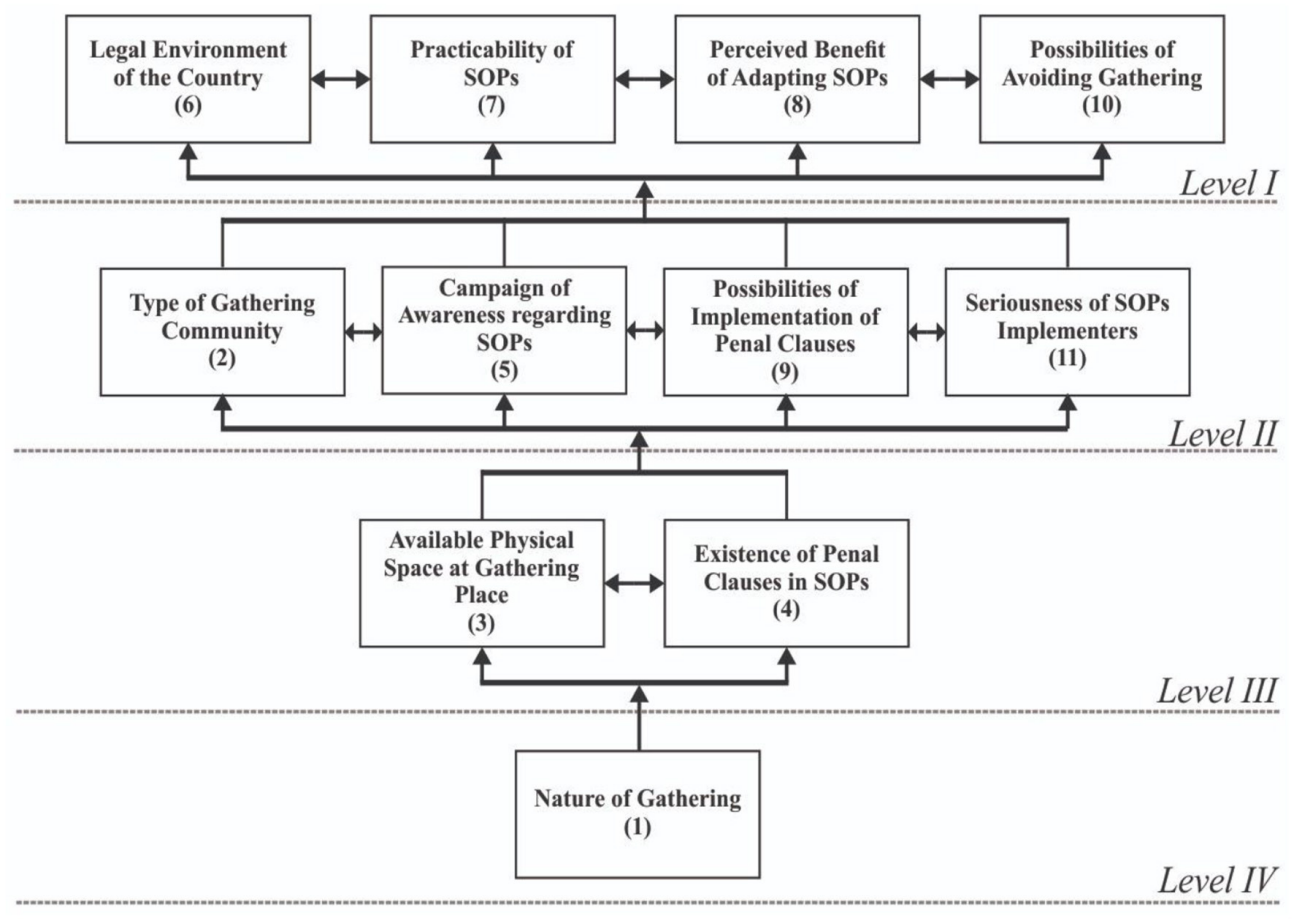
ISM model.

The model captured on the diagonal of the conical matrix was converted into a graphical representation as Figure 1.

Close observation of the model revealed that the determinants coded as 6, 7, 8, and 10 fall on *Level I*. The determinants coded as 2, 5, 9, and 11 fall at *Level II*. The determinants coded as 3 and 4 fall at *Level III* and the determinant coded as 1 falls at *Level IV*. The model was generated from the conical matrix in the classical format of bottom-top. The bottom of the model is considered to be the most critical and the top to be the comparatively least critical. In this way, the factors that occupy the bottom of the model are critical determinants of the adoption of SOPs in mass gatherings. The factors (determinants in this case) occupying the middle of the model are moderately severe or critical, whereas those that occupy the top are considered as less important or severe. Further analysis of the model in this regard is presented in the following sections with corroboration of the results through MICMAC.

### MICMAC Analysis

**Figure 2:**
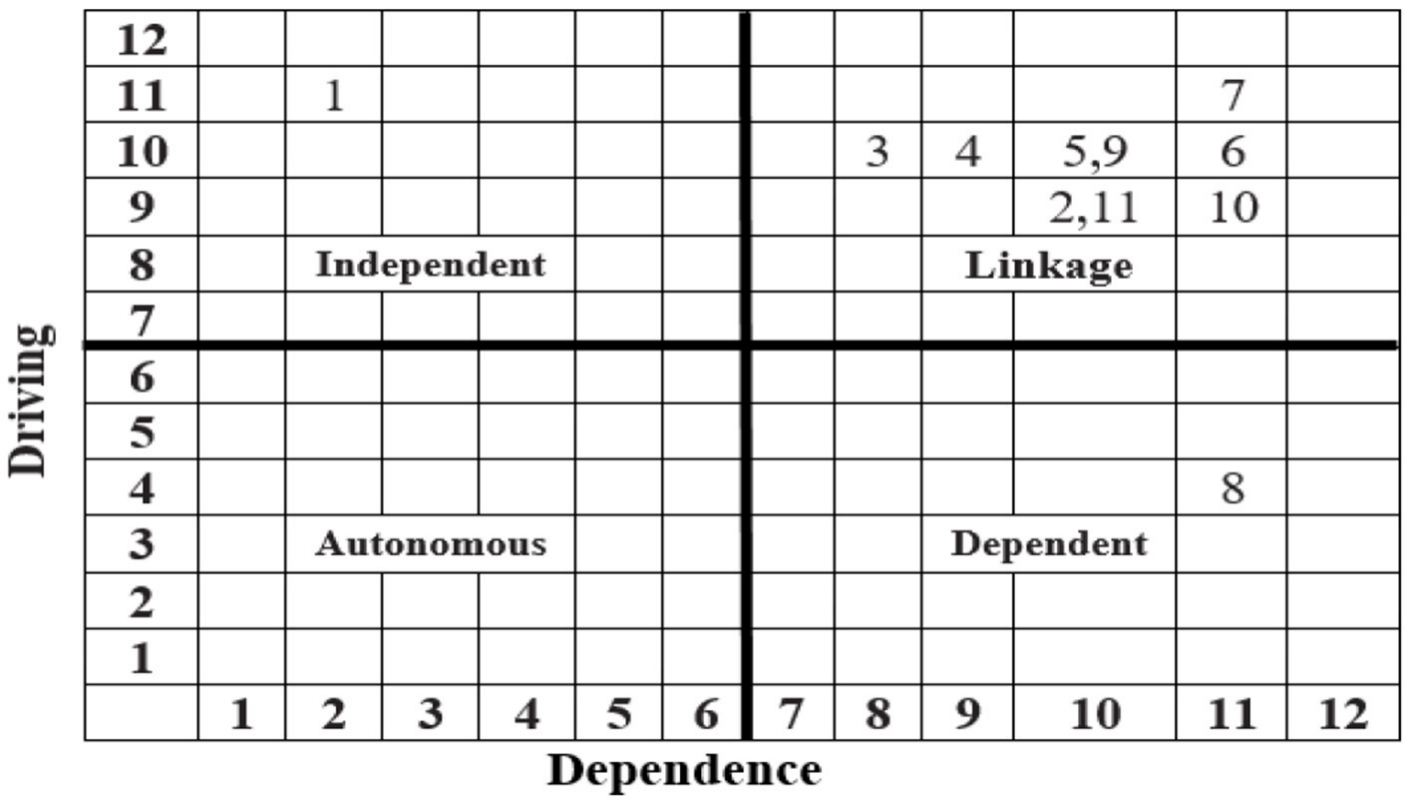
Driving-dependence diagram.

In order to corroborate the findings of the ISM method, MICMAC analysis (Godet, 1986) was performed, as shown in Figure 2. Following the scale centric approach, we divided the Cartesian plane into four quadrants i.e., independent, autonomous, dependent, and linkage.

Close observation of the driving-dependence diagram (Figure 2) revealed that the determinant coded as 8 is categorized in the dependent quadrant, the determinant coded as 1 is categorized in the independent quadrant, and determinants coded as 2, 3, 4, 5, 6, 7, 9, 10, and 11 are categorized in the linkage quadrant, while no determinant falls in autonomous.

### Results

The objective of the study is to analyze the determinants of the effectiveness of SOPs in mass gatherings for containing COVID-19. During the course of the study relevant determinants were identified, regarding which the data was collected and ISM and MICMAC were applied. The results of the literature review revealed that there are a total of 11 important determinants of the effective implementation of SOPs for containing COVID-19 (Table 1). The results of the ISM show that determinants legal environment of the country (6), practicability of SOPs (7), perceived benefit of adapting SOPs (8), and possibilities of avoiding gathering (10) fall at *Level I* (top level). The determinants that occupy the top level of the model are considered less critical. The factors at the top of the model are dependent or driven by other factors. The determinants type of gathering community (2), campaign of awareness regarding SOPs (5), possibilities of implementation of penal clauses (9), and seriousness of SOPs implementers (11) fall at *Level II* (middle level), and the determinants available physical space at gathering place (3) and existence of penal clauses in SOPs (4) fall at *Level III* (middle level). The determinants that occupy the middle of the model are moderately critical and are mediators or moderators. The determinant nature of gathering (1) falls at *Level IV* (bottom level). The determinants that occupy the bottom of the model are considered as the most critical factors, and they are independent and drivers and they affect the upper levels critically. The bottom of the model deserves the immediate attention of stakeholders, particularly policymakers. In the current study, “nature of gathering” is the most critical factor, deserving immediate attention of health policymakers. Results of MICMAC are as follows: *Dependent:* the factors (in this case the determinants) that fall in the dependent quadrant have a high dependence power but a low driving power, which means the dependent factors are driven by other factors. The determinant “perceived benefit of adapting SOPs (8)” falls in the dependent quadrant. *Independent:* The independent factors are those factors that fall in the independent quadrant. They have a high driving power but a low dependence power. These are important, critical, and need immediate attention. If these issues are addressed they can affect the other factors accordingly. The determinant “nature of gathering” (1) falls in the independent quadrant. *Autonomous:* the factors that fall in the autonomous quadrant are considered to be independent of the system. They deserve to be eliminated from the system but since they have a few powerful links they are included in the study. In ISM models, most of the time they stand disconnected from the others. No determinant falls in the autonomous quadrant, so all the determinants in the study are highly connected to the system and are very much part of the system under study. *Linkage:* the factors that fall in the linkage quadrant are considered ambivalent, agile, unsettled, or unbalanced. If most of the factors are classified into the quadrant namely ‘Linkage' in MICMAC analysis, it necessarily means that the system under study is not settled and it is trying to make some sense. The determinants **“**type of gathering community (2),” “available physical space at gathering place (3),” “existence of penal clauses in SOPs (4),” “campaign of awareness regarding SOPs (5),” “legal environment of the country (6),” “practicability of SOPs (7),” “possibilities of implementation of penal clauses (9),” “possibilities of avoiding gathering (10),” and “seriousness of SOPs implementers (11)” fall in the linkage quadrant. The overall results of the study show that “nature of gathering (1)” is the key determinant. The results are summarized in Table 11.

**Table 11:** Summarized and juxtaposed results of the study.

**Result of literature review**		**Results of MICMAC**				**Results of ISM**	**Comments**
**Code**	**Issue**	**Driving**	**Dependence**	**Effectiveness**	**Cluster**	**Level**	
***1***	*Nature of gathering*	***11***	*2*	*9*	*Independent*	*IV*	*Key factor*
**2**	Type of gathering community	**9**	10	−1	Linkage	II	
**3**	Available physical space at gathering place	**10**	8	2	Linkage	III	
**4**	Existence of penal clauses in SOPs	**10**	9	1	Linkage	III	
**5**	Campaign of awareness regarding SOPs	**10**	10	0	Linkage	II	
**6**	Legal environment of the country	**10**	11	−1	Linkage	I	
**7**	Practicability of SOPs	**11**	11	0	Linkage	I	
**8**	Perceived benefit of adapting SOPs	**4**	11	−7	Dependent	I	
**9**	Possibilities of implementation of penal clauses	**10**	10	0	Linkage	II	
**10**	Possibilities of avoiding gathering	**9**	11	−2	Linkage	I	
**11**	Seriousness of SOPs implementers	**9**	10	−1	Linkage	II	

The results of MICMAC analysis affirmed the results of the ISM. According to the ISM, “nature of gathering (1)” (gray highlighted in Table 11) occupies the bottom of the model and is therefore important/independent, and this result is also confirmed by MICMAC analysis as this factor is categorized in the independent quadrant, having a high driving power and low dependence power. Other results are also almost the same in both methodologies.

### Discussion

Since the main objective of the study is to analyze the determinants of the effectiveness of the SOPs for the COVID-19 pandemic in mass gathering, it is a fundamentally important and research worthy topic in today's world. Therefore, it is worthwhile to discuss the results of the study in contrast with the current literature. We have identified representative determinants, form them into a hierarchy, imposed a structure on interactive relations among them, and categorized them into different quadrants based on their driving-dependence. In the literature, there are few studies that also have addressed this issue, partially or remotely. A comparative analysis of the studies closer to the current study is represented in Table 12.

**Table 12:** Comparison with studies from literature.

**Sr**.	**Study**	**Country**	**Focus**	**Variables**	**Methodology**	**Result**
**1**	Current study	Pakistan	Determinants of effectiveness of SOPs in mass gatherings for containing COVID-19	11	Empirical study using ISM and MICMAC methods	Nature of gathering is key determinant.
**2**	Yezli and Khan (2020)	Saudi Arabia	Implementation of “Jeddah Tool” that offers a health risk assessment framework	–	Theoretical study	“Jeddah Tool” a practical and standardized guide to strategic all-hazard HRA in mass gatherings.
**3**	El Alaoui (2020)	Japan	Estimation of infections in mass gatherings	–	Example using probability theory	Method of making approximation.
**4**	Memish et al. (2020)	Saudi Arabia	Suspending mass gatherings	–	Theoretical study	Allowing mass gatherings is potential to endanger for attendees.

Yezli and Khan (2020) conducted a theoretical study in the Kingdom of Saudi Arabia and emphasized the use of the “Jeddah Tool” as a standardized guide for handling hazards. It is a suggestive study without any empirical evidence. El Alaoui (2020) conducted a study in Japan focusing on the possibilities of estimation of infections in mass gatherings. It uses probability theory and illustrates the approximation of infections as a result of mass gatherings. Memish et al. (2020) advocated for suspending mass gatherings in view of the possible danger of rapid transmission of COVID-19. It is a theoretical study that builds logical arguments. Although these studies directly examine the phenomenon of mass gathering, the current study addresses altogether the different aspects of mass gathering. This study is different from them in context, in methodology (design, population, sampling, analysis, etc.), and in results.

## Conclusion

This study has a fundamental importance for planning and preparing for mass gathering events while ensuring the minimum risk of COVID-19 transmission. The study aims to analyze the determinants of the effectiveness of SOPs in mass gatherings for containing COVID-19. The problem of unclear determinants of the implementation of the SOPs for preventing coronavirus, especially in mass gatherings, is studied in this research. The overall design of the study involves a literature review, data collection survey, structural modeling, and analysis. As a methodology, discussion of the literature review is used to identify the determinants, ISM is used to develop and analyze a structural model, and MICMAC is used for corroborating the results of the ISM/classification of determinants. The results of the literature review reveal that there are a total of 11 important determinants of effective implementation of SOPs of COVID-19 containment (Table 1). The results of the ISM show that the determinants “legal environment of the country (6),” “practicability of SOPs (7),” “perceived benefit of adapting SOPs (8),” and “possibilities of avoiding gathering (10)” fall at *Level I*, the determinants “type of gathering community (2),” “campaign of awareness regarding SOPs (5),” “possibilities of implementation of penal clauses (9),” and “seriousness of SOPs implementers (11)” fall at *Level II*, tge determinants “available physical space at gathering place (3),” and “existence of penal clauses in SOPs (4)” fall at *Level III* and the determinant “nature of gathering (1)” falls at *Level IV*. The results of MICMAC show that the determinant “perceived benefit of adapting SOPs (8)” falls in the dependent quadrant, the determinant “nature of gathering (1)” falls in the independent quadrant, no determinants fall in the autonomous quadrant, and the determinants **“**type of gathering community (2),” “available physical space at gathering place (3),” “existence of penal clauses in SOPs (4),” “campaign of awareness regarding SOPs (5),” “legal environment of the country (6),” “practicability of SOPs (7),” “possibilities of implementation of penal clauses (9),” “possibilities of avoiding gathering (10),” and “seriousness of SOPs implementers (11)” fall in the linkage quadrant. The overall results of the study show that “nature of gathering (1”) is the key determinant.

### Contribution of the Study

The study contributed a list of determinants, an ISM model, a MICMAC diagram, and simplified information about the inter-relationship of the determinants toward the contemporary literature.

### Practical and Theoretical Implications

This study is useful for local authorities to regulate mass gatherings in local communities since it develops their understanding of the inter-determinant relationships. It is useful for organizers and participants (attendees/visitors) of mass gatherings because it provides a lot of information for understanding the implications of following/not-following the SOPs of COVID-19 containment. It is helpful to health officials/regulators since it identifies key determinants, provides information about the relationships of these determinants, and prioritizes them on the basis of their importance of handling. It is also helpful for researchers by offering research frameworks since it categorizes the determinants into independent, dependent, autonomous, and mediators/moderators. It also proposes many relationships that can be hypothesized and tested statistically by future researchers. The study is useful for the community at large because it provides a lot of new information and understanding of the phenomenon under study.

### Limitations of the Study and Directions for Future Studies

There are certain limitations of the study as well. First, it uses a qualitative approach to address the issue; therefore, future studies should use quantitative approaches to address the issue. Second, the data has been collected from Pakistan (a Muslim country); therefore, the results of the study should accordingly be generalized and interpreted. In this context, future studies may be conducted in different countries. Third, a limited list of determinants was investigated since the literature was not very rich in this regard; therefore, future studies should enrich the list and replicate the study. Fourth, the data has been collected from a limited number of experts (focus group considered to be representative), it is recommended that in the future the data should be collected from a statistical population in order to augment or enhance the results of the study.

### Policy Recommendations

Keeping in view the results of the study (“nature of gathering” being the key determinant and most of the other determinants being agile, unbalanced, or unsettled) it is recommended for policymakers to devise gathering specific SOPs and carefully evaluate the effects of unbalanced determinants in order to achieve the desired results of precautionary measures of COVID-19 pandemic.

## Data Availability Statement

The raw data supporting the conclusions of this article will be made available by the authors, without undue reservation.

## Author Contributions

AB: initiated the idea and worked on ISM analysis. AN: worked on the relevant literature of the topic. TQ and Z-u-RR: collected the data and performed the analyses. MS: worked on the write up. AM: conceptualization. MS: supervision of research process/review process and funding acquisition. MS, MZS, and AM: writing (review & editing). All authors contributed to the article and approved the submitted version.

## Funding

This research is supported by Lund University, Lund, Sweden to the extent of APC therefore we acknowledge the support. We also acknowledge the respondents who participated as experts.

## Conflict of Interest

The authors declare that the research was conducted in the absence of any commercial or financial relationships that could be construed as a potential conflict of interest.

## Publisher's Note

All claims expressed in this article are solely those of the authors and do not necessarily represent those of their affiliated organizations, or those of the publisher, the editors and the reviewers. Any product that may be evaluated in this article, or claim that may be made by its manufacturer, is not guaranteed or endorsed by the publisher.

## Appendix

**APPENDIX 1:** Matrix indicating paired relationships.

**Code**	**Determinants**	**1**	**2**	**3**	**4**	**5**	**6**	**7**	**8**	**9**	**10**	**11**
**1**	Nature of gathering											
**2**	Type of gathering community											
**3**	Available physical space at gathering place											
**4**	Existence of penal clauses in SOPs											
**5**	Campaign of awareness regarding SOPs											
**6**	Legal environment of the country											
**7**	Practicability of SOPs											
**8**	Perceived benefit of adapting SOPs											
**9**	Possibilities of implementation of penal clauses											
**10**	Possibilities of avoiding gathering											
**11**	Seriousness of SOPs implementers											

## References

[B1] Ahmed Q. A. Memish Z. A. (2020). The cancellation of mass gatherings (MGs)? Decision making in the time of COVID-19. Travel Med. Infect. Dis. 34, 101631. 10.1016/j.tmaid.2020.101631 32184129 PMC7102544

[B2] Akon M. S. Rahman M. (2020). Reshaping the global order in the post covid-19 era: a critical analysis. Chin. J. Int. Rev. 2, 2050006. 10.1142/S2630531320500067

[B3] Alshammari T. M. Altebainawi A. F. Alenzi K. A. (2020). Importance of early precautionary actions in avoiding the spread of COVID-19: Saudi Arabia as an example. Saudi Pharmaceut. J. 28, 898–902. 10.1016/j.jsps.2020.05.005 32641902 PMC7242187

[B4] Al-Tawfiq J. A. Memish Z. A. Zumla A. (2021). Mass religious gatherings events and COVID-19-easing of COVID-19 restrictions and a staged approach to scaling up the umrah pilgrimage. Travel Med. Infect. Dis. 40, 101986. 10.1016/j.tmaid.2021.101986 33567359

[B5] Atique S. Itumalla R. (2020). Hajj in the time of COVID-19. Infect. Dis. Health 25, 219–221. 10.1016/j.idh.2020.04.001 32305323 PMC7158770

[B6] Bhosale V. A. Kant R. (2016). An integrated ISM fuzzy MICMAC approach for modelling the supply chain knowledge flow enablers. Int. J. Product. Res. 54, 7374–7399. 10.1080/00207543.2016.1189102

[B7] Carlin P. R. Minard P. Simon D. H. Wing C. (2021). Effects of large gatherings on the COVID-19 epidemic: evidence from professional and college sports large gatherings' effects on COVID-19. Econ. Hum. Biol. 2021, 101033. 10.1016/j.ehb.2021.101033 34298460 PMC8643424

[B8] Chakraborty I. Maity P. (2020). COVID-19 outbreak: migration, effects on society, global environment and prevention. Sci. Total Environ. 728, 138882. 10.1016/j.scitotenv.2020.138882 32335410 PMC7175860

[B9] Ebrahim S. H. Memish Z. A. (2020). COVID-19–the role of mass gatherings. Travel Med. Infect. Dis. 34, 101617. 10.1016/j.tmaid.2020.101617 32165283 PMC7102534

[B10] El Alaoui A. (2020). How countries of south mitigate COVID-19: models of morocco and Kerala, India.

[B11] Furuse Y. (2021a). Risk at mass-gathering events and the usefulness of complementary events during COVID-19 pandemic. J. Infect. 82, e20–e21. 10.1016/j.jinf.2020.11.040 33271175 PMC9190236

[B12] Furuse Y. (2021b). Genomic sequencing effort for SARS-CoV-2 by country during the pandemic. Int. J. Infect. Dis. 103, 305–307. 10.1016/j.ijid.2020.12.034 33333251 PMC7832795

[B13] Godet M. (1986). Introduction to la prospective: seven key ideas and one scenario method. Futures 18, 134–157. 10.1016/0016-3287(86)90094-7

[B14] Hashim H. T. Babar M. S. Essar M. Y. Ramadhan M. A. Ahmad S. (2021). The Hajj and COVID-19: how the pandemic shaped the world's largest religious gathering. Am. J. Trop. Med. Hyg. 104, 797–799. 10.4269/ajtmh.20-1563 33432907 PMC7941851

[B15] Hoang V. T. Gautret P. Memish Z. A. Al-Tawfiq J. A. (2020). Hajj and Umrah mass gatherings and COVID-19 infection. Curr. Trop. Med. Rep. 7, 133–140. 10.1007/s40475-020-00218-x 33169095 PMC7609349

[B16] Hsu C. Y. Chen Y. M. Su C. W. Ku M. S. Kim Y. Jensen T.. (2021). Preparedness for containing COVID-19 outbreak in mass religious gathering with non-pharmaceutical interventions (NPIs). J. Formos. Med. Assoc. 120, S57–S68. 10.1016/j.jfma.2021.04.017 34119393 PMC8173477

[B17] Hughes D. Saw R. Perera N. K. P. Mooney M. Wallett A. Cooke J.. (2020). The Australian Institute of Sport framework for rebooting sport in a COVID-19 environment. J. Sci. Med. Sport 23, 639–663. 10.1016/j.jsams.2020.05.004 32451268 PMC7200343

[B18] Jena J. Sidharth S. Thakur L. S. Pathak D. K. Pandey V. C. (2017). Total interpretive structural modeling (TISM): approach and application. J. Adv. Manage. Res. 14, 162–181. 10.1108/JAMR-10-2016-0087

[B19] Kumar S. Luthra S. Haleem A. (2013). Customer involvement in greening the supply chain: an interpretive structural modeling methodology. J. Ind. Eng. Int. 9, 6. 10.1186/2251-712X-9-6

[B20] Li G. Huang D. Sun C. Li Y. (2019). Developing interpretive structural modeling based on factor analysis for the water-energy-food nexus conundrum. Sci. Total Environ. 651, 309–322. 10.1016/j.scitotenv.2018.09.188 30240915

[B21] Li M. Yang J. (2014). Analysis of interrelationships between critical waste factors in office building retrofit projects using interpretive structural modelling. Int. J. Construct. Manage. 14, 15–27. 10.1080/15623599.2013.875270

[B22] Lohaus D. Habermann W. (2019). Presenteeism: a review and research directions. Hum. Resour. Manage. Rev. 29, 43–58. 10.1016/j.hrmr.2018.02.010

[B23] Majumdar A. Sinha S. K. (2019). Analyzing the barriers of green textile supply chain management in Southeast Asia using interpretive structural modeling. Sustain. Product. Consump. 17, 176–187. 10.1016/j.spc.2018.10.005

[B24] Mat N. F. C. Edinur H. A. Razab M. K. A. A. Safuan S. (2020). A single mass gathering resulted in massive transmission of COVID-19 infections in Malaysia with further international spread. J. Travel Med. 27, 1–4. 10.1093/jtm/taaa059 32307549 PMC7188142

[B25] McCloskey B. Zumla A. Ippolito G. Blumberg L. Arbon P. Cicero A.. (2020). Mass gathering events and reducing further global spread of COVID-19: a political and public health dilemma. Lancet 395, 1096–1099. 10.1016/S0140-6736(20)30681-4 32203693 PMC7138150

[B26] Memish Z. A. Ahmed Q. A. Schlagenhauf P. Doumbia S. Khan A. (2020). No time for dilemma: mass gatherings must be suspended. Lancet 395, 1191–1192. 10.1016/S0140-6736(20)30754-6 32240624 PMC7146682

[B27] Memish Z. A. Steffen R. White P. Dar O. Azhar E. I. Sharma A.. (2019). Mass gatherings medicine: public health issues arising from mass gathering religious and sporting events. Lancet 393, 2073–2084. 10.1016/S0140-6736(19)30501-X 31106753 PMC7159069

[B28] Mubarak N. (2020). Corona and clergy-the missing link for effective social distancing in Pakistan: time for some unpopular decisions. Int. J. Infect. Dis. 95, 431–432. 10.1016/j.ijid.2020.04.067 32360938 PMC7192112

[B29] Mubarak N. Zin C. S. (2020). Religious tourism and mass religious gatherings-the potential link in the spread of COVID-19. Current perspective and future implications. Travel Med. Infect. Dis. 36, 101786. 10.1016/j.tmaid.2020.101786 32531422 PMC7282735

[B30] Niazi A. A. K. Qazi T. F. Basit A. (2019). an interpretive structural model of barriers in implementing Corporate Governance (CG) in Pakistan. Glob. Region. Rev. 4, 359–375. 10.31703/grr.2019(IV-I)0.39

[B31] Nyasulu J. C. Y. Munthali R. J. Nyondo-Mipando A. L. Pandya H. Nyirenda L. Nyasulu P. S.. (2021). COVID-19 pandemic in Malawi: did public sociopolitical events gatherings contribute to its first-wave local transmission? Int. J. Infect. Dis. 106, 269–275. 10.1016/j.ijid.2021.03.055 33771674 PMC8751974

[B32] Piovani D. Christodoulou M. N. Hadjidemetriou A. Pantavou K. Zaza P. Bagos P. G.. (2021). Effect of early application of social distancing interventions on COVID-19 mortality over the first pandemic wave: an analysis of longitudinal data from 37 countries. J. Infect. 82, 133–142. 10.1016/j.jinf.2020.11.033 33275956 PMC7706420

[B33] Qazi T. F. Niazi A. A. K. Hameed R. Basit A. (2020). How they get stuck? Issues of women entrepreneurs: an interpretive structural modeling approach. Paradigms 14, 73–79.

[B34] Quadri S. A. (2020). COVID-19 and religious congregations: implications for spread of novel pathogens. Int. J. Infect. Dis. 96, 219–221. 10.1016/j.ijid.2020.05.007 32389851 PMC7204705

[B35] Rehman H. Ahmad M. I. (2020). COVID-19: a wreak havoc across the globe. Arch. Physiol. Biochem. 2020, 1–13. 10.1080/13813455.2020.1797105 32730095

[B36] Rodriguez-Morales A. J. Sah R. Paniz-Mondolfi A. (2020). The holy week 2020 and the beginning of COVID-19 epidemics in Latin America. Travel Med. Infect. Dis. 2020, 101633. 10.1016/j.tmaid.2020.101633 32205270 PMC7142679

[B37] Shaukat M. Z. Niazi A. A. K. Qazi T. F. Basit A. (2021). Analyzing the underlying structure of online teaching during the COVID-19 pandemic period: an empirical investigation of issues of students. Front. Psychol. 12, 605138. 10.3389/fpsyg.2021.605138 33935860 PMC8084101

[B38] Sokhna C. Goumballa N. Bassene H. Parola P. Gautret P. (2021). The grand magal of touba was spared by the COVID-19 pandemic. Int. J. Infect. Dis. 105, 470–471. 10.1016/j.ijid.2021.01.006 33434665 PMC9183244

[B39] Sonis J. D. Black L. Baugh J. Benzer T. I. Hayes B. D. Raja A. S.. (2020). Leveraging existing quality improvement communication strategies during the COVID-19 crisis. Am. J. Emerg. Med. 38, 1523–1524. 10.1016/j.ajem.2020.04.021 32312576 PMC7151532

[B40] Sushil S. (2012). Interpreting the interpretive structural model. Glob. J. Flex. Syst. Manage. 13, 87–106. 10.1007/s40171-012-0008-3

[B41] Teixeira S. C. (2020). COVID-19 and mass gatherings: emerging and future implications of the Brazilian carnival for public health. Public Health 187, 62. 10.1016/j.puhe.2020.08.003 32927289 PMC7483076

[B42] VanderWeele T. J. (2020). Love of neighbor during a pandemic: navigating the competing goods of religious gatherings and physical health. J. Relig. Health 59, 2196. 10.1007/s10943-020-01031-6 32405925 PMC7218704

[B43] Warfield J. N. (1973). Binary matrices in system modeling. IEEE Trans. Syst. Man Cybern. 5, 441–449. 10.1109/TSMC.1973.4309270

[B44] Warfield J. N. (1974). Toward interpretation of complex structural models. IEEE Trans. Syst. Man Cybern. 5, 405–417. 10.1109/TSMC.1974.4309336

[B45] World Health Organization (2021). (WHO). Available online at: https://covid19.who.int/ (accessed August 7, 2021).

[B46] Xiao L. (2018). Analyzing consumer online group buying motivations: an interpretive structural modeling approach. Telemat. Inform. 35, 629–642. 10.1016/j.tele.2018.01.010

[B47] Yezli S. Khan A. (2021). COVID-19 pandemic: it is time to temporarily close places of worship and to suspend religious gatherings. J. Travel Med. 28, taaa065. 10.1093/jtm/taaa065 32339236 PMC7197566

[B48] Yezli S. Khan A. A. (2020). The Jeddah tool: a health risk assessment framework for mass gatherings. Saudi Med. J. 41, 121. 10.15537/smj.2020.2.24875 32020143 PMC7841630

